# P021: Effect of urinary tract infections at multiresistant bacteria (MRB) in hospital of Dakar

**DOI:** 10.1186/2047-2994-2-S1-P21

**Published:** 2013-06-20

**Authors:** R Ka, NMD Badiane, A Ndir, KL Onanga, NMD Badiane, ML Dia, R Diagne, B Ndoye, AI Sow, M Seydi

**Affiliations:** 1 Department of Bacteriology, CHU Fann, Senegal; 2 Department of Infectious Diseases, CHU Fann, Senegal; 3 PRONALIN, Ministère of Health and Social Action, Dakar, Senegal; 4 Department of Bacteriology, Fann Hospital, Dakar, Senegal

## Introduction

The control of the spread of BMR in health facilities is a national priority in Senegal.

## Objectives

Our work aims to study the incidence of urinary tract infections due to BMR in a university hospital.

## Methods

Microbiological monitoring was conducted in three inpatient wards of a university hospital from April to October 2012 and concerned only diagnostic urinalysis.

## Results

During the study period, 123 patients were followed up and 79 urine samples were made corresponding to 56.4% of all diagnostic samples. The average age of patients was 55 ± 21.22 and sex ratio of 0.97. Thirty-nine percent of patients were admitted with a neurological disorder. These patients were referred by another health facility (56.3%) came from home (34.4%) or had undergone internal transfer (9.4%). The attack rate of urinary tract infections was 3.96. These urinary tract infections were of nosocomial acquisition in 56 cases (78.9%) with an average delay of 12.75 days. The bacteria isolated were Enterobacteriaceae 61 (77.2%), non-fermenting Gram-negative bacilli 14 (17.7%) and staphylococci 4 (5.1%). In order of frequency, these were Escherichia coli 25 (31.6%), Klebsiella pneumoniae 21 (26.6%), Pseudomonas 8 (10.2%) and Enterobacter 5 (6.3%). We found 32 strains (40.5%) producing extended-spectrum beta-lactamase (ESBL) and 2 strains (2.5%) of Staphylococcus aureus resistant to methicillin (MRSA). From urinary tract infections associated with ESBL Enterobacteriaceae urinary catheter was potentially the source of infection in 18 cases. BMR attack rate was 1.80 per 100 admissions and the incidence rate of 1.99 BMR for 1000 days patients. To wane, 18 patients or 30% died.

## Conclusion

The decrease in the incidence of nosocomial urinary tract infections must be accompanied by antibiotic stewardship and a strict hygiene policy.

## Disclosure of interest

None declared

